# Application of an efficient Bayesian discretization method to biomedical data

**DOI:** 10.1186/1471-2105-12-309

**Published:** 2011-07-28

**Authors:** Jonathan L Lustgarten, Shyam Visweswaran, Vanathi Gopalakrishnan, Gregory F Cooper

**Affiliations:** 1 Department of Biomedical Informatics and the Intelligent Systems Program, University of Pittsburgh, Suite M-183 Vale, Parkvale Building, 200 Meyran Avenue, Pittsburgh, PA 15260, USA; 2 University of Pennsylvania School of Veterinary Medicine, 3800 Spruce Street, Philadelphia, PA 19104, USA

## Abstract

**Background:**

Several data mining methods require data that are discrete, and other methods often perform better with discrete data. We introduce an efficient Bayesian discretization (EBD) method for optimal discretization of variables that runs efficiently on high-dimensional biomedical datasets. The EBD method consists of two components, namely, a Bayesian score to evaluate discretizations and a dynamic programming search procedure to efficiently search the space of possible discretizations. We compared the performance of EBD to Fayyad and Irani's (FI) discretization method, which is commonly used for discretization.

**Results:**

On 24 biomedical datasets obtained from high-throughput transcriptomic and proteomic studies, the classification performances of the C4.5 classifier and the naïve Bayes classifier were statistically significantly better when the predictor variables were discretized using EBD over FI. EBD was statistically significantly more stable to the variability of the datasets than FI. However, EBD was less robust, though not statistically significantly so, than FI and produced slightly more complex discretizations than FI.

**Conclusions:**

On a range of biomedical datasets, a Bayesian discretization method (EBD) yielded better classification performance and stability but was less robust than the widely used FI discretization method. The EBD discretization method is easy to implement, permits the incorporation of prior knowledge and belief, and is sufficiently fast for application to high-dimensional data.

## Background

With the advent of high-throughput techniques, such as DNA microarrays and mass spectrometry, transcriptomic and proteomic studies are generating an abundance of high-dimensional biomedical data. The analysis of such data presents significant analytical and computational challenges, and increasingly data mining techniques are being applied to these data with promising results [1-4]. A typical task in such analysis, for example, entails the learning of a mathematical model from gene expression or protein expression data that predicts well a phenotype, such as disease or health. In data mining, such a task is called classification and the model that is learned is termed a classifier. The variable that is predicted is called the target variable (or simply the target), which in statistical terminology is referred to as the response or the dependent variable. The features used in the prediction are called the predictor variables (or simply the predictors), which are referred to as the covariates or the independent variables in statistical terminology.

A large number of data mining methods have been developed for classification; several of these methods are unable to use continuous data and require discrete data [1-3]. For example, most rule learning methods that induce sets of IF-THEN rules and several of the popular methods that learn Bayesian networks require data that are discrete. Some methods that accept continuous data, as for example methods that learn classification trees, discretize the data internally during learning. Other methods, such as the naïve Bayes classifier, that accept both continuous and discrete data, may perform better with discrete data [3,4]. A variety of discretization methods have been developed for converting continuous data to discrete data [5-11], and one that is commonly used is Fayyad and Irani's (FI) discretization method [9].

In this paper, we present an efficient Bayesian discretization method and evaluate its performance on several high-dimensional transcriptomic and proteomic datasets, and we compare its performance to that of the FI discretization method. The remainder of this paper is structured as follows. The next section provides some background on discretization and briefly reviews the FI discretization method. The results section describes the efficient Bayesian discretization (EBD) method and gives the results of an evaluation of EBD and FI on biomedical transcriptomic and proteomic datasets. The final section discusses the results and draws conclusions.

### Discretization

Numerical variables may be *continuous *or *discrete*. A continuous variable is one which takes an infinite number of possible values within a range or an interval. A discrete variable is one which takes a countable number of distinct values. A discrete variable may take few values or a large number of values. Discretization is a process that transforms a variable, either discrete or continuous, such that it takes a fewer number of values by creating a set of contiguous intervals (or equivalently a set of cut points) that spans the range of the variable's values. The set of intervals or the set of cut points produced by a discretization method is called a discretization.

Discretization has several advantages. It broadens the range of classification algorithms that can be applied to datasets since some algorithms cannot handle continuous attributes. In addition to being a necessary pre-processing step for classification methods that require discrete data, discretization has been shown to increase the accuracy of some classifiers, increase the speed of classification methods especially on high-dimensional data, and provide better human interpretability of models such as IF-THEN rule sets [8,10,11]. The impact of discretization on the performance of classifiers is not only due to the conversion of continuous values to discrete ones, but also due to filtering of the predictor variables [4]. Variables that are discretized to a single interval are effectively filtered out and discarded by classification methods since they are not predictive of the target variable. Due to redundancy and noise in the predictor variables in high-dimensional transcriptomic and proteomic data, such filtering of variables has the potential to improve classification performance. Even classification methods like Support Vector Machines and Random Forests that handle continuous variables directly and are robust to high dimensionality of the data may benefit from discretization [4]. The main disadvantage of discretization is the loss of information entailed in the process that has the potential to reduce performance of classifiers if the information loss is relevant for classification. However, this theoretical concern may or may not be a practical one, depending on the particular machine-learning situation.

Discretization methods can be classified as unsupervised or supervised. Unsupervised methods do not use any information about the target variable in the discretization process while supervised methods do. Examples of unsupervised methods include the Equal-Width method, which partitions the range of variable's values into a user-specified number of intervals and the Equal-Frequency method, which partitions the range of variable's values into a user-specified fraction of instances per interval. Compared to unsupervised methods, supervised methods tend to be more sophisticated and typically yield classifiers that have superior performance [8,10,11]. Most supervised discretization methods consist of a score to measure the goodness of a set of intervals (where goodness is a measure of how well the discretized predictor variable predicts the target variable), and a search method to locate a good-scoring set of intervals in the space of possible discretizations. The commonly used FI method is an example of a supervised method.

A second way to categorize discretization methods is as univariate versus multivariate methods. Univariate methods discretize a continuous-valued variable independently of all other predictor variables in the data, while multivariate methods take into consideration the possible interactions of the variable being discretized with the other predictor variables. Multivariate methods are rarely used in practice since they are computationally more expensive than univariate methods and have been developed for specialized applications [12,13]. The FI discretization method is a typical example of a univariate method.

We now introduce terminology that will be useful for describing discretization. Let *D *be a dataset of *n *instances consisting of the list ((*X*_1_, *Z*_1_), (*X*_2_, *Z*_2_), ..., (*X*_*k*_, *Z*_*k*_), ..., (*X*_*n*_, *Z*_*n*_)) that is sorted in ascending order of *X*_*k*_, where *X*_*k *_is a real value of the predictor variable *X *and *Z*_*k *_is the associated integer value of the target variable *Z*. For example, suppose that the predictor variable represents the expression level of a gene that takes real values in the range 0 to 5.0 and the target variable represents the phenotype that takes the values: *healthy *or *diseased *(*Z *= 0 or *Z *= 1, respectively). Then, an example dataset *D *is ((1.2, 0), (1.4, 0), (1.6, 0), (3.7, 1), (3.9, 1), (4.1, 1)). Let *S*_*a, b *_be a list of the first elements of *D*, starting at the *a*^th ^pair in *D *and ending at the *b*^th ^pair. Thus, for the above example, *S*_4, 6 _= (3.7, 3.9, 4.1). For brevity, we denote by *S *the list *S*_1, *n*_. Let *T*_*b *_be a set that represents a discretization of *S*_1, *b*_. For the above example of *D*, a possible 2-interval discretization is *T*_6 _= {*S*_1, 3_, *S*_4, 6_} = {(1.2, 1.4, 1.6), (3.7, 3.9, 4.1)}. Equivalently, this 2-interval discretization denotes a cut point between 1.6 and 3.7, and typically the mid-point is chosen, which is 2.65 in this example. Thus, all values below 2.65 are considered as a single discrete value and all values equal or greater than 2.65 are considered another discrete value. For brevity, we denote by *T *a discretization *T*_*n *_of *S*.

### Fayyad and Irani's (FI) Discretization Method

Fayyad and Irani's discretization method is a univariate supervised method that is widely used and has been cited over 2000 times according to Google Scholar^1^. The FI method consists of i) a score that is the entropy of the target variable induced by the discretization of the predictor variable, and ii) a greedy search method that recursively discretizes each partition at a cutpoint that minimizes the joint entropy of the two resulting subintervals until a stopping criterion based on the minimum description length (MDL) is met.

For a list *S*_*a, b *_derived from a predictor variable *X *and a target variable *Z *that takes *J *values, the entropy *Ent*(*S*_*a, b*_) is defined as:(1)

where, *P*(*Z *= *z*_*j*_) is the proportion of instances in *S*_*a, b *_where the target takes the value *z*_*j*_. The entropy of *Z *can be interpreted as a measure of its uncertainty or disorder. Let a cutpoint *C *split the list *S*_*a, b *_into the lists *S*_*a, c *_and *S*_*c *+ 1, *b *_to create a 2-interval discretization {*S*_*a, c*_, *S*_*c *+ 1, *b*_}. The entropy *Ent*(*C*; *S*_*a, b*_) induced by *C *is given by:(2)

where, |*S*_*a, b*_| is the number of instances in *S*_*a, b*_, |*S*_*a, c*_| is the number of instances in *S*_*a, c*_, and |*S*_*c *+ 1, *b*_| is the number of instances in *S*_*c *+ 1, *b*_. The FI method selects the cut point *C *from all possible cut points that minimizes *Ent*(*C*; *S*_*a, b*_) and then recursively selects a cut point in each of the newly created intervals in a similar fashion. As partitioning always decreases the entropy of the resulting discretization, the process of introducing cut points is terminated by a MDL-based stopping criterion. Intuitively, minimizing the entropy results in intervals where each interval has a preponderance of one value for the target.

Overall, the FI method is very efficient and runs in *O*(*n log n*) time, where *n *is the number of instances in the dataset. However, since it uses a greedy search method, it does not examine all possible discretizations and hence is not guaranteed to discover the optimal discretization, that is, the discretization with the minimum entropy.

### Minimum Optimal Description Length (MODL) Discretization Method

To our knowledge, the closest prior work to the EBD algorithm, which is introduced in this paper, is the MODL algorithm that was developed by Boulle [5]. MODL is a univariate, supervised, discretization algorithm. Both MODL and EBD use dynamic programming to search over discretization models that are scored using a Bayesian measure. EBD differs from MODL in two important ways. First, MODL assumes uniform prior probabilities over the discretization, whereas EBD allows an informative specification of both structure and parameter priors, as discussed in the next section. Thus, although EBD can be used with uniform prior probabilities as a special case, it is not required to do so. If we have background knowledge or beliefs that may influence the discretization process, EBD provides a way to incorporate them into the discretization process.

Second, the MODL optimal discretization algorithm has a run time that is *O*(*n*^3^), whereas the EBD optimal discretization algorithm has a run time of *O*(*n*^2^), where *n *is the number of instances in the dataset. In essence, EBD uses a more efficient form of dynamic programming, than does MODL. Their difference in computational time complexity can have significant practical consequences in terms of which datasets are feasible to use. A dataset with, for example, 10,000 instances might be practical to use in performing discretization using EBD, but not using MODL.

While heuristic versions of MODL have been described [5], which give up optimality guarantees in order to improve computational efficiency, and heuristic versions of EBD could be developed that further decrease its time complexity as well, the focus of the current paper is on optimal discretization.

In the next section, we introduce the EBD algorithm and then describe an evaluation of it on a set of bioinformatics datasets.

## Results

### An Efficient Bayesian Discretization Method

We now introduce a new supervised univariate discretization method called efficient Bayesian discretization (EBD). EBD consists of i) a Bayesian score to evaluate discretizations, and ii) a dynamic programming search method to locate the optimal discretization in the space of possible discretizations. The dynamic programming method examines all possible discretizations and hence is guaranteed to discover the optimal discretization, that is, the discretization with the highest Bayesian score.

### Bayesian Score

We first describe a discretization model and define its parameters. As before, let *X *and *Z *denote the predictor and target variables, respectively, let *D *be a dataset of *n *instances consisting of the list ((*X*_1_, *Z*_1_), (*X*_2_, *Z*_2_), ..., (*X*_*k*_, *Z*_*k*_), ..., (*X*_*n*_, *Z*_*n*_)), as described above, and let *S *denote a list of the first elements of *D*. A discretization model *M *is defined as:

where, *W *is the number of intervals in the discretization, *T *is a discretization of *S*, and Θ is defined as follows. For a specified interval *i*, the distribution of the target variable *P*(*Z *| *W *= *i*) is modeled as a multinomial distribution with the parameters {*θ*_*i *1_,*θ*_*i*2_,...,*θ*_*ij*_,...,*θ*_*iJ*_} where *j *indexes the distinct values of *Z*. Considering all the intervals, Θ = {*θ*_*ij*_} over 1 ≤ *i *≤ *I *and 1 ≤ *j *≤ *J *and Θ specifies all the multinomial distributions for all the intervals in *M*. Given data *D*, EBD computes a Bayesian score for all possible discretizations of *S *and selects the one with the highest score.

We now derive the Bayesian score used by EBD to evaluate a discretization model *M*. The posterior probability *P*(*M *| *D*) of *M *is given by Bayes rule as follows:(3)

where *P*(*M*) is the prior probability of *M, P*(*D *| *M*) is the marginal likelihood of the data *D *given *M*, and *P*(*D*) is the probability of the data. Since *P*(*D*) is the same for all discretizations, the Bayesian score evaluates only the numerator on the right hand side of Equation 3 as follows:(4)

The marginal likelihood *P*(*D *| *M*) in Equation 4 is derived using the following equation:(5)

where Θ are the parameters of the multinomial distributions as defined above. Equation 5 has a closed-form solution under the following assumptions: (1) the values of the target variable were generated according to i.i.d. sampling from *P*(*Z *| *W *= *i*), which is modeled with a multinomial distribution, (2) the distribution *P*(*Z *| *W *= *i*) is modeled as being independent of the distribution *P*(*Z *| *W *= *h*) for all values of *i *and *h *such that *i *≠ *h*, (3) for all values *i*, prior belief about the distribution *P*(*Z *| *W *= *i*) is modeled with a Dirichlet distribution with hyperparameters *α*_*ij*_, and (4) there are no missing data. The closed-form solution to the marginal likelihood is given by the following expression [14,15]:(6)

where Γ(·) is the gamma function, *n*_*i *_is the number of instances in the interval *i, n*_*ij *_is the number of instances in the interval *W*_*i *_that have target-value *j, α*_*ij *_are the hyperparameters in a Dirichlet distribution which define the prior probability over the *θ*_*ij *_parameters, and . The hyperparameters can be viewed as prior counts, as for example from a previous (or a hypothetical) dataset of instances in the interval *i *that belong to the value *j*. For the experiments described in this paper, we set all the *α*_*ij *_to 1, which can be shown to imply that *a priori *we assume all possible distributions of *P*(*Z *| *W *= *i*) to be equally likely, for each interval *i*.^2 ^If all *α*_*ij *_= 1, then all *α*_*i *_= *J*. With these values for the hyperparameters, and using the fact that Γ(*n*) = (*n*-1)!, Equation 6 becomes the following:(7)

The term *P*(*M*) in Equation 4 specifies the prior probability on the number of intervals and the location of the cut points in the discretization model *M*; we call these the *structure priors*. The structure priors may be chosen to penalize complex discretization models with many intervals to prevent overfitting. In addition to the structure priors, the marginal likelihood *P*(*D *| *M*) includes a specification of the prior probabilities on the multinomial distribution of the target variable in each interval; we call these the *parameter priors*. In Equation 6, the alphas specify the parameter priors.

The prior probability *P*(*M*) is modeled as follows. Let *X*_*k *_denote a real value of the predictor variable, as described above, and *Z*_*k *_denote the associated integer value of the target variable. Let *Prior*(*k*) be the prior probability of there being at least one cut point between *X*_*k *_and *X*_*k *+ 1_. In the Methods section, we describe the use of a Poisson distribution with mean λ to implement *Prior*(*k*), where λ is a structure prior parameter. Consider the prior probability for an interval *i *that represents the sequence  in a discretization model *M*. In general, we assume that the prior probability for interval *i *is independent of the prior probabilities for the other intervals in *M*. The prior probability for interval *i *in terms of the *Prior *function is defined as follows:(8)

Expression 8 gives the prior probability that no cut points are present between any consecutive pairs of values of *X *in the sequence  and at least one cut point is present between the values  and . Using the above notation and assumptions, and substituting Equations 7 and 8 into Equation 4, we obtain the specialized EBD score:(9)

The above score assumes that the *n *values of *X *in the dataset *D *are all distinct. However, the implementation described below easily relaxes that assumption.

### Dynamic Programming Search

**Figure 1:**
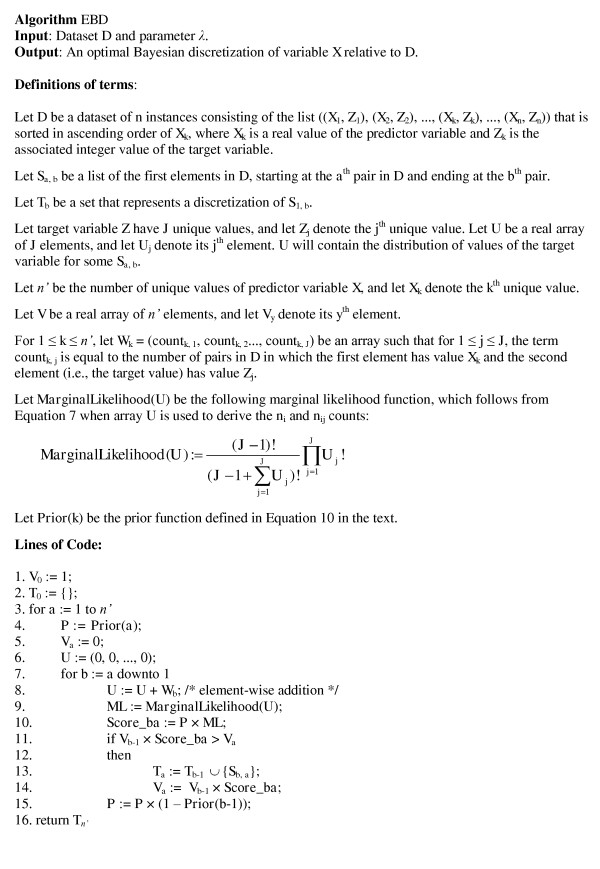
**Pseudocode for the efficient Bayesian discretization (EBD) method**. The EBD method uses dynamic programming and runs in *O*(*n*^2^) time as indicated by the two *for *loops (*n *is the number of instances in the dataset).

The EBD method finds the discretization that maximizes the score given in Equation 9 using dynamic programming to search the space of possible discretizations. The pseudocode for the EBD search method is given in Figure 1. It is globally optimal in that it is guaranteed to find the discretization with the highest score. Additional details about the search method used by EBD and its time complexity are provided in the Methods section.

The number of possible discretizations for a predictor variable *X *in a dataset with *n *instances is 2^*n-*1^, and this number is typically too large for each discretization to be evaluated in a brute force manner. The EBD method addresses this problem by the use of dynamic programming that at every stage uses previously computed optimal solutions to subproblems. The use of dynamic programming reduces considerably the number of possible discretizations that have to be evaluated explicitly without sacrificing the ability to identify the optimal discretization.

**Figure 2:**
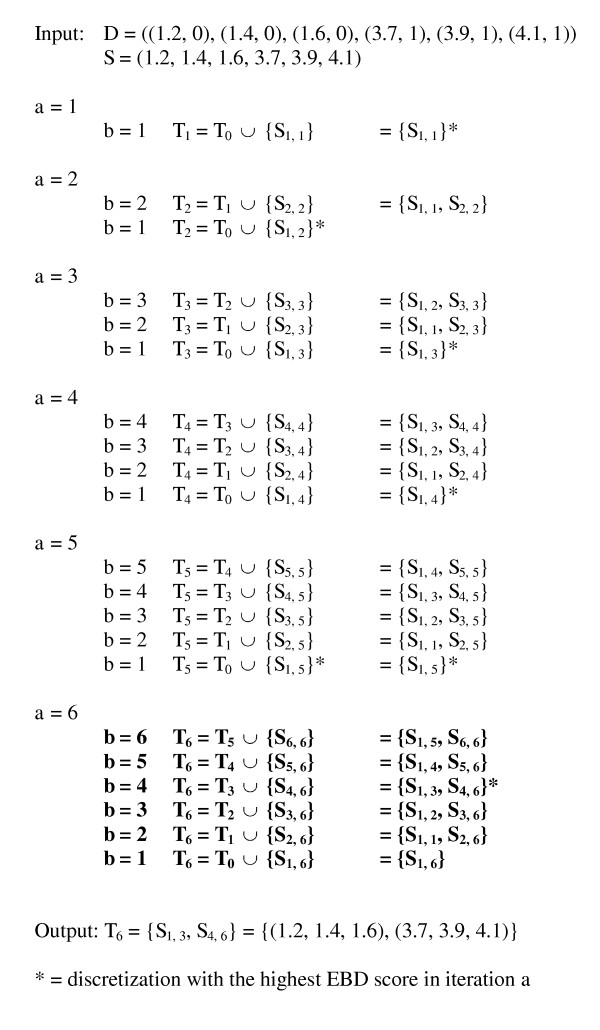
**An example of the application of the efficient Bayesian discretization (EBD) method**. This example shows the progression of the EBD method when applying the pseudocode given in Figure 1 to the dataset of six instances that is introduced in the main text. An asterisk denotes the discretization with the highest EBD score in a given iteration, as indexed by *a*. There are 2^5 ^= 32 possible discretizations for a dataset of six instances; for this dataset EBD explicitly evaluates only the 6 discretizations shown in bold font.

An example of the application of the EBD method on the example dataset *D *= ((1.2, 0), (1.4, 0), (1.6, 0), (3.7, 1), (3.9, 1), (4.1, 1)) is given in Figure 2. Although there are 2^5 ^= 32 possible discretizations for a dataset of six instances, as in this example, EBD explicitly evaluates only 6 of them in determining the highest scoring discretization.

As described in the Methods section, the EBD algorithm runs in *O*(*n*^2^) time, where *n *is the number of instances of a predictor *X*. Although EBD is slower than FI, it is still feasible to apply EBD to high-dimensional data with a large number of variables.

### Evaluation of the Efficient Bayesian Discretization (EBD) Method

We evaluated the EBD method and compared its performance to the FI method on 24 biomedical datasets (see Table 1) using five measures: accuracy, area under the Receiver Operating Characteristic curve (AUC), robustness, stability, and the mean number of intervals per variable (a measure of model complexity). The last three measures evaluate the discretized predictors directly while the first two measures evaluate the performance of classifiers that are learned from the discretized predictors. We performed this comparison using the FI method, because it is so commonly used (1) in practice and (2) as a standard algorithmic benchmark for discretization methods.

**Table 1:** Description of datasets

Dataset	Dataset name	Type	P/D	#t	#n	#V	M
1	Alon et al.	T	D	2	61	6,584	0.651
2	Armstrong et al.	T	D	3	72	12,582	0.387
3	Beer et al.	T	P	2	86	5,372	0.795
4	Bhattacharjee et al.	T	D	7	203	12,600	0.657
5	Bhattacharjee et al.	T	P	2	69	5,372	0.746
6	Golub et al.	T	D	2	72	7,129	0.653
7	Hedenfalk et al.	T	D	2	36	7,464	0.500
8	Iizuka et al.	T	P	2	60	7,129	0.661
9	Khan et al.	T	D	4	83	2,308	0.345
10	Nutt et al.	T	D	4	50	12,625	0.296
11	Pomeroy et al.	T	D	5	90	7,129	0.642
12	Pomeroy et al.	T	P	2	60	7,129	0.645
13	Ramaswamy et al.	T	D	29	280	16,063	0.100
14	Rosenwald et al.	T	P	2	240	7,399	0.574
15	Staunton et al.	T	D	9	60	7,129	0.145
16	Shipp et al.	T	D	2	77	7,129	0.747
17	Su et al.	T	D	13	174	12,533	0.150
18	Singh et al.	T	D	2	102	10,510	0.510
19	Veer et al.	T	P	2	78	24,481	0.562
20	Welsch et al.	T	D	2	39	7,039	0.878
21	Yeoh et al.	T	P	2	249	12,625	0.805
22	Petricoin et al.	P	D	2	322	11,003	0.784
23	Pusztai et al.	P	D	3	159	11,170	0.364
24	Ranganathan et al.	P	D	2	52	36,778	0.556

In the Type column, T denotes *transcriptomic *and P denotes *proteomic*. In the P/D column, P denotes *prognostic *and D denotes *diagnostic*. #t is the number of values of the target variable and #n is the number of instances in the dataset. #V is the number of predictor variables. M is the proportion of the data that has the majority target value.

For computing the evaluation measures we performed 10 × 10 cross-validation (10-fold cross-validation done ten times to generate a total of 100 training and test folds). For a pair of training and test folds, we learned a discretization model for each variable (using either FI or EBD) for the training fold only and applied the intervals from the model to both the training and test folds to generate the discretized variables. For the experiments, we set *λ*, which is user specified parameter introduced in Figure 1 and in Equation 10 (see the Methods section) to be 0.5. The parameter *λ *is the expected number of cut points in the discretization of the variables in the domain. Our previous experience with discretizing some of the datasets used in the experiments with FI indicated that the majority of the variables in these datasets have 1 or 2 intervals (that correspond to 0 or 1 cut points). We chose *λ *to be 0.5 as the average of 0 and 1 cut points.

We used two classifiers in our experiments, namely, C4.5 and naïve Bayes (NB). C4.5 is a popular tree classifier that accepts both continuous and discrete predictors and has the advantage that the classifier can be interpreted as a set of rules. The NB classifier is simple, efficient, robust, and accepts both continuous and discrete predictors. It assumes that the predictors are conditionally independent of each other given the target value. Given an instance, it applies Bayes theorem to compute the probability distribution over the target values. This classifier is very effective when the independence assumptions hold in the domain; however, even if these assumptions are violated, the classification performance is often excellent, even when compared to more sophisticated classifiers [16].

Accuracy is a widely used measure of predictive performance (see the Methods section). The mean accuracies for EBD and FI for C4.5 and NB are given in Table 2. EBD has higher mean accuracy on 17 datasets for each of C4.5 and NB, respectively. FI has higher mean accuracy on 4 datasets and 3 datasets for C4.5 and NB, respectively. EBD and FI have the same mean accuracy on 4 datasets and 3 datasets for C4.5 and NB, respectively. Overall, EBD shows an increase in accuracy of 2.02% and 0.76% for C4.5 and NB, respectively. This increased performance is statistically significant at the 5% significance level on the Wilcoxon signed rank test for both C4.5 and NB.

**Table 2:** Accuracies for the EBD and FI discretization methods

Classifier	C4.5		NB	
**Dataset**	**EBD (SEM)**	**FI (SEM)**	**EBD (SEM)**	**FI (SEM)**

1	100.00% (0.00)	100.00% (0.00)	93.33% (0.93)	93.33% (0.85)
2	**86.43**% (0.79)	84.62% (0.77)	**93.03**% (1.06)	92.12% (0.94)
3	**78.61**% (1.30)	64.23% (1.72)	81.53% (1.11)	81.53% (1.02)
4	**88.62**% (0.66)	84.38% (0.67)	**75.43**% (0.83)	72.76% (0.79)
5	**59.04**% (1.72)	56.33% (1.93)	**71.19**% (0.92)	69.78% (1.13)
6	**96.67**% (1.10)	95.67% (0.82)	**82.32**% (1.17)	80.28% (1.51)
7	94.46% (1.03)	94.46% (1.03)	97.32% (0.84)	97.32% (0.84)
8	**60.00**% (2.08)	50.00% (2.03)	**72.33**% (1.42)	70.82% (1.49)
9	**83.61**% (1.28)	81.29% (0.97)	91.94% (0.91)	**93.67**% (0.72)
10	**68.00**% (1.98)	66.54% (1.21)	**76.00**% (1.65)	71.76% (1.32)
11	**77.67**% (1.30)	72.44% (0.91)	**75.53**% (1.33)	73.81% (1.11)
12	55.83% (2.14)	**59.58**% (2.12)	**63.33**% (1.81)	61.67% (1.84)
13	**58.92**% (0.86)	57.14% (0.96)	**50.36**% (0.84)	49.32% (0.88)
14	58.75% (0.91)	**62.33**% (1.01)	**58.33**% (1.04)	57.65% (1.09)
15	**54.94**% (0.72)	54.20% (0.74)	**55.34**% (1.70)	53.86% (1.07)
16	**72.43**% (1.32)	71.25% (1.45)	**86.22**% (1.41)	85.45% (1.22)
17	**70.06**% (0.94)	68.96% (1.17)	**82.81**% (0.79)	81.78% (1.42)
18	81.21% (0.58)	**83.78**% (0.68)	83.76% (0.91)	**89.76**% (0.75)
19	**74.12**% (1.32)	72.22% (1.21)	**85.12**% (1.09)	84.19% (1.31)
20	59.45% (2.08)	59.45% (2.08)	100.00% (0.00)	100.00% (0.00)
21	62.32% (1.54)	**65.24**% (1.43)	**78.23**% (0.59)	76.23% (0.54)
22	**73.22**% (0.78)	69.78% (1.21)	**78.23**% (0.77)	77.23% (0.78)
23	**73.32**% (0.92)	68.49% (0.98)	46.22% (0.98)	**48.55**% (0.87)
24	**76.12**% (1.32)	73.04% (1.72)	**83.32**% (1.65)	80.12% (1.23)

Average	**73.49**% (2.07)	71.48% (2.12)	**77.55**% (2.65)	76.79% (2.32)

Accuracies for EBD and FI discretization methods are obtained from the application of C4.5 and NB classifiers to the discretized variables. The mean and the standard error of the mean (SEM) for the accuracy for each dataset is obtained by 10 × 10 cross-validation. For each dataset, the higher accuracy is shown in bold font and equal accuracies are underlined.

The AUC is a measure of the discriminative performance of a classifier that accounts for datasets that have a highly skewed distribution over the target variable (see the Methods section). The mean AUCs for EBD and FI for C4.5 and NB are given in Table 3. For C4.5, EBD has higher mean AUC on 17 datasets, FI has higher mean AUC on 5 datasets, and both discretization methods have the same mean AUC on 2 datasets. For NB, EBD has higher mean AUC than FI on 16 datasets, lower mean AUC on 6 datasets, and the same mean AUC on two datasets. Overall, EBD shows an improvement in AUC of 1.07% and 1.12% for C4.5 and NB, respectively, and both increases in AUC are statistically significant at the 5% level on the Wilcoxon signed rank test.

**Table 3:** AUCs for the EBD and FI discretization methods

Classifier	C4.5		NB	
**Dataset**	**EBD**	**FI**	**EBD**	**FI**

1	98.00% (0.06)	98.00% (0.06)	**69.32**% (0.88)	66.79% (0.92)
2	**73.19**% (1.08)	69.37% (1.22)	78.58% (1.87)	**79.96**% (1.98)
3	**57.24**% (1.88)	55.42% (1.65)	**56.08**% (1.70)	54.16% (1.92)
4	68.37% (1.27)	**69.43**% (0.95)	58.12% (1.08)	**59.72**% (1.17)
5	**55.21**% (1.12)	54.38% (1.44)	**56.87**% (1.41)	53.91% (1.09)
6	**61.54**% (0.63)	60.11% (0.95)	**88.21**% (0.66)	86.38% (0.86)
7	88.45% (1.42)	88.45% (1.42)	91.35% (0.76)	91.35% (0.76)
8	54.11% (1.12)	**55.49**% (0.89)	58.76% (0.85)	**59.61**% (0.76)
9	**88.34**% (1.32)	86.90% (1.41)	**87.65**% (1.18)	84.28% (1.12)
10	**76.45**% (0.68)	74.30% (0.81)	**85.44**% (0.99)	82.59% (1.04)
11	**68.25**% (0.71)	66.12% (0.61)	**72.38**% (1.01)	70.74% (0.98)
12	**56.65**% (1.21)	55.14% (1.06)	**57.89**% (0.95)	53.72% (0.86)
13	70.45% (0.87)	**73.18**% (0.65)	69.89% (0.71)	**71.55**% (0.75)
14	**56.32**% (1.12)	55.16% (0.98)	54.42% (0.98)	**55.12**% (0.96)
15	**76.12**% (0.87)	73.49% (1.01)	89.45% (0.89)	**91.27**% (0.56)
16	**82.21**% (1.31)	80.06% (1.12)	**82.86**% (1.17)	80.11% (1.09)
17	78.65% (1.41)	**80.15**% (1.32)	**78.14**% (1.12)	75.98% (1.24)
18	**94.75**% (0.87)	92.31% (0.90)	**96.12**% (0.65)	94.19% (0.72)
19	**76.31**% (1.25)	74.23% (1.14)	**82.42**% (1.03)	81.16% (1.24)
20	94.12% (1.19)	**95.43**% (1.21)	100.00% (0.00)	100.00% (0.00)
21	**54.24**% (0.75)	52.13% (0.46)	**55.09**% (0.43)	54.92% (0.65)
22	**64.18**% (0.94)	60.65% (0.98)	**64.87**% (0.89)	64.25% (0.71)
23	**83.24**% (0.76)	81.56% (0.79)	**77.23**% (0.97)	76.17% (0.88)
24	**80.86**% (1.01)	80.21% (0.89)	**84.72**% (0.89)	81.21% (0.77)

Average	**73.22**% (1.89)	72.15% (1.77)	**74.83**% (1.43)	73.71% (1.24)

AUCs for EBD and FI discretization methods are obtained from the application of C4.5 and NB classifiers to the discretized variables. The mean and the standard error of the mean (SEM) for the AUC for each dataset is obtained by 10 × 10 cross-validation. For each dataset, the higher AUC is shown in bold font and equal AUCs are underlined.

Robustness is the ratio of the accuracy on the test dataset to that on the training dataset expressed as a percentage (see the Methods section). The mean robustness for EBD and FI for C4.5 and NB are given in Table 4. For C4.5, EBD has higher mean robustness on 10 datasets, FI has higher mean robustness on 11 datasets, and both have equivalent mean robustness on three datasets. For NB, EBD has better performance than FI on 9 datasets, worse performance on 13 datasets, and similar performance on two datasets. Overall, EBD shows a small decrease in mean robustness of 0.26% and 0.68% for C4.5 and NB, respectively, that are not statistically significant at the 5% level on the Wilcoxon signed rank test.

**Table 4:** Robustness for the EBD and FI discretization methods

Classifier	C4.5		NB	
**Dataset**	**EBD (SEM)**	**FI (SEM)**	**EBD (SEM)**	**FI (SEM)**

1	100.00% (0.00)	100.00% (0.00)	94.94% (0.89)	94.94% (0.97)
2	**90.64**% (0.77)	87.69% (0.86)	94.36% (0.98)	**95.17**% (1.05)
3	**70.78**% (2.00)	53.57% (2.10)	**82.44**% (1.10)	81.69% (1.14)
4	84.18% (0.76)	**85.87**% (0.77)	**90.10**% (1.09)	75.91% (1.09)
5	49.83% (2.01)	**53.08**% (2.18)	69.97% (1.20)	**86.88**% (1.12)
6	**83.58**% (1.34)	80.58% (1.42)	**97.76**% (1.12)	95.89% (0.92)
7	92.50% (1.18)	92.50% (1.18)	96.67% (0.86)	**97.27**% (0.86)
8	**55.50**% (2.16)	55.11% (2.06)	70.94% (1.48)	**71.67**% (1.43)
9	**90.61**% (0.95)	87.16% (0.99)	**98.98**% (0.74)	96.08% (0.94)
10	**75.10**% (1.48)	68.65% (1.39)	74.35% (2.05)	**76.93**% (1.81)
11	70.36% (0.95)	**70.47**% (0.93)	78.25% (1.22)	**82.52**% (1.20)
12	57.82% (2.22)	**61.04**% (2.21)	63.47% (1.87)	**65.94**% (1.88)
13	66.12% (0.39)	**66.96**% (0.37)	**64.89**% (1.05)	50.83% (1.02)
14	57.47% (0.94)	**64.13**% (1.06)	67.01% (1.08)	**69.18**% (1.08)
15	**54.94**% (0.72)	54.20% (0.74)	54.16% (1.75)	**61.60**% (1.70)
16	73.17% (1.66)	**77.17**% (1.79)	**92.57**% (1.39)	84.11% (1.38)
17	82.71% (1.35)	**87.43**% (1.21)	**88.25**% (1.56)	85.49% (1.60)
18	79.38% (0.57)	**82.65**% (0.57)	88.91% (0.72)	**91.81**% (0.83)
19	73.00% (1.48)	**79.00**% (1.30)	85.55% (1.31)	**85.89**% (1.29)
20	58.75% (2.09)	58.75% (2.08)	100.00% (0.00)	100.00% (0.00)
21	55.18% (1.26)	**62.23**% (1.13)	77.01% (0.60)	**76.10**% (0.57)
22	**72.53**% (0.96)	66.84% (1.03)	**90.87**% (0.89)	81.15% (0.93)
23	**78.16**% (1.04)	76.07% (0.99)	**77.79**% (1.67)	52.49% (1.73)
24	**75.00**% (1.78)	70.00% (1.75)	80.33% (1.64)	**99.86**% (1.70)

Average	72.55% (2.81)	**72.81**% (2.76)	81.72% (2.92)	**82.40**% (2.59)

The mean and the standard error of the mean (SEM) for robustness for each dataset is obtained by 10 × 10 cross-validation. For each dataset, the higher robustness value is shown in bold font and equal robustness values are underlined.

Stability quantifies how different training datasets affect the variables being selected (see the Methods section). The mean stabilities for EBD and FI are given in Table 5. Overall, EBD has higher stability than FI, but only at an overall average of 0.02, which nevertheless is statistically significant at the 5% significance level on the Wilcoxon signed rank test.

**Table 5:** Stabilities for the EBD and FI discretization methods

Dataset	EBD	FI
1	**0.83**	0.80
2	0.84	0.84
3	**0.68**	0.67
4	0.82	**0.84**
5	**0.54**	0.50
6	0.79	**0.80**
7	**0.81**	0.79
8	**0.58**	0.55
9	**0.84**	0.82
10	**0.83**	0.81
11	**0.80**	0.75
12	0.50	0.50
13	0.76	**0.78**
14	0.53	0.53
15	**0.65**	0.59
16	**0.80**	0.79
17	**0.81**	0.75
18	0.76	**0.82**
19	**0.75**	0.69
20	**0.88**	0.84
21	**0.60**	0.42
22	**0.86**	0.85
23	0.89	**0.94**
24	0.59	**0.61**

Average	**0.74**	0.72

The mean stability for each dataset is obtained by 10 × 10 cross-validation. For each dataset, the higher stability value is shown in bold font and equal stability values are underlined.

Table 6 gives the mean number of intervals obtained by EBD and FI. The first column gives for each dataset the proportion of predictor variables that were discretized into a single interval, that is, there were no cut points. Such predictors are considered uninformative and are not used for learning a classifier. The second column gives for each dataset the mean number of intervals among those predictors that were discretized to more than one interval. The third column reports the mean number of intervals over all predictors, including intervals that contain no cut points. Overall, the application of EBD resulted in more predictors with more than one interval, relative to the application of FI, by an overall average of 9%. Also, the mean number of intervals per predictor was greater for EBD than for FI, but this difference was not statistically significant at the 5% level on the Wilcoxon signed rank test. This implies that while the average for the EBD complexity is slightly greater (1.27 versus 1.16 intervals per predictor), overall, EBD and FI are similar in terms of complexity of the discretizations produced.

**Table 6:** Mean number of intervals per predictor variable for the EBD and FI discretization methods

	Mean fraction of predictors with 1 interval		Mean # of intervals per predictor with >1 interval		Mean # of intervals per predictor	
**Dataset**	**EBD**	**FI**	**EBD**	**FI**	**EBD**	**FI**

1	0.81	**0.84**	**2.02**	2.01	1.15	**1.16**
2	0.47	**0.61**	**2.06**	2.04	**1.48**	1.41
3	0.91	**0.96**	**2.02**	2.01	**1.05**	1.04
4	0.18	**0.28**	**2.20**	2.16	**1.91**	1.84
5	0.97	**0.99**	2.03	**2.04**	1.01	1.01
6	0.87	**0.89**	2.01	2.01	**1.13**	1.11
7	0.82	**0.86**	**2.02**	2.01	1.13	**1.14**
8	0.97	**0.98**	2.02	2.02	1.01	**1.02**
9	0.54	**0.76**	2.11	**2.12**	**1.42**	1.27
10	0.38	**0.65**	2.06	2.06	**1.53**	1.37
11	0.51	**0.77**	2.06	**2.10**	**1.41**	1.25
12	0.98	**0.99**	2.02	2.02	1.01	1.01
13	0.05	**0.90**	**2.57**	2.10	**2.39**	1.11
14	0.98	**0.99**	**2.03**	2.02	1.01	1.01
15	0.70	**0.98**	2.08	**2.12**	**1.20**	1.02
16	0.75	**0.87**	2.01	2.01	1.12	**1.13**
17	0.76	**0.85**	2.04	2.04	1.16	1.16
18	0.17	**0.78**	**2.31**	2.13	**1.99**	1.25
19	0.87	**0.94**	**2.05**	2.02	1.06	1.06
20	0.81	**0.85**	2.02	**2.10**	1.15	**1.17**
21	0.97	**0.99**	2.01	**2.02**	1.01	1.01
22	0.82	**0.84**	2.14	2.14	1.16	**1.18**
23	0.93	**0.97**	2.01	**2.02**	**1.05**	1.03
24	0.92	**0.95**	2.06	**2.02**	1.04	**1.05**

Average	0.76	**0.85**	**2.08**	2.06	**1.27**	1.16

The mean fraction of predictor variables discretized to one interval (no cut points), the mean number of intervals for predictor variables discretized to more than one interval (at least one cut point), and the mean number of intervals for all predictor variables for each dataset is obtained by 10-fold cross-validation done ten times. For each dataset, the higher value is shown in bold font and equal values are underlined.

The results of the statistical comparison of the EBD and FI discretization methods using the Wilcoxon paired samples signed rank test are given in Table 7. As shown in the table, the accuracy and AUC of C4.5 and NB classifiers were statistically significantly better at the 5% level when the predictor variables were discretized using EBD over FI. EBD was statistically significantly more stable to the variability of the datasets than FI. However, EBD was less robust, though not statistically significantly so, than FI and produced slightly more complex discretizations than FI.

**Table 7:** Statistical comparison of EBD and FI discretization methods

Evaluation Measure	Method	Mean (SEM)	Difference of Means	Z statistic (*p*-value)
C4.5 Accuracy	EBD	73.49% (2.07)	2.01	2.219
[0%, 100%]	FI	71.48% (2.12)		(**0.026**)

C4.5 AUC	EBD	73.22% (1.89)	1.07	2.732
[50%, 100%]	FI	72.15% (1.77)		(**0.007**)

C4.5 Robustness	EBD	72.55% (2.81)	-0.26	-0.261
[0%, ∞]	FI	72.81% (2.76)		(0.794)

NB Accuracy	EBD	77.55% (2.65)	0.76	2.080
[0%, 100%]	FI	76.79% (2.32)		(**0.038**)

NB AUC	EBD	74.83% (1.43)	1.11	2.711
[0%, 100%]	FI	73.71% (1.24)		(**0.007**)

NB Robustness	EBD	81.72% (2.92)	-0.68	-0.016
[50%, ∞]	FI	82.40% (2.59)		(0.987)

Stability	EBD	0.74 (0.025)	0.02	1.972
[0, 1]	FI	0.72 (0.029)		(**0.049**)

Mean # of intervals per predictor	EBD	1.27 (0.074)	0.11	1.686
[1, *n*]	FI	1.16 (0.038)		(0.092)

In the first column the range of a measure is given in square brackets where *n *is the number of instances in the dataset. In the last column the number on top in the last column is the Z statistic and the number at the bottom is the corresponding *p*-value. On all performance measures, except for the mean number of intervals per predictor, the Z statistic is positive when EBD performs better than FI. The two-tailed *p*-values of 0.05 or smaller are in bold, indicating that EBD performed statistically significantly better at that level.

### Running Times

We conducted the experiments on an AMD X2 4400 + 2.2 GHz personal computer with 2GB of RAM that was running Windows XP. For the 24 datasets included in our study, on average to discretize all the predictor variables in a dataset, EBD took 20 seconds per training fold while FI took 5 seconds per training fold.

## Discussion

We have developed an efficient Bayesian discretization method that uses a Bayesian score to evaluate a discretization and employs dynamic programming to efficiently search and identify the optimal discretization. We evaluated the performance of EBD on several measures and compared it to the performance of FI. Table 8 shows the number of wins, draws and losses when comparing EBD to FI on accuracy, AUC, stability and robustness. On both accuracy and AUC, which are measures of discrimination performance, EBD demonstrated statistically significant improvement over FI. EBD was more stable than FI, which indicates that EBD is less sensitive to the variability of the training datasets. FI was moderately better in terms of robustness, but not statistically significantly so. On average, EBD produced slightly more intervals per predictor variable, as well as a greater proportion of predictors that had more than one interval. Thus, EBD produced slightly more complex discretizations than FI.

**Table 8:** Summary of wins, draws and losses of EBD versus FI

Evaluation Measure	Wins	Draws	Losses
C4.5 Accuracy	17	3	4
C4.5 AUC	17	2	5
C4.5 Robustness	10	3	11
NB Accuracy	17	4	3
NB AUC	16	2	6
NB Robustness	9	2	13
Stability	15	3	6

Number of wins, draws and losses on accuracy, AUC, robustness and stability for EBD and FI.

A distinctive feature of EBD is that it allows the specification of parameter and structure priors. Although we used non-informative parameter priors in the evaluation reported here, EBD readily supports the use of informative prior probabilities, which enables users to specify background knowledge that can influence how a predictor variable is discretized. The alpha parameters in Equation 6 are the parameter priors. Suppose there are two similar biomedical datasets A and B containing the same variables, but different populations of individuals, and we are interested in discretizing the variables. The data in A could provide information for defining the parameter priors in Equation 6 before its application to the data in B. There is a significant amount of flexibility in defining this mapping for using data in a similar (but not identical) biomedical dataset to influence the discretization of another dataset. The lambda parameter in Equation 10 (described in the Methods section) allows the user to provide a structure prior. This is where prior knowledge might be particularly helpful by specifying (probabilistically) the expected number of cut points per predictor variable. Although we have presented a structure prior that is based on a Poisson distribution, the EBD algorithm can be readily adapted to use other distributions. In doing so, the main assumption is that a structure prior of an interval can be composed as a product of the structure priors of its subintervals.

The running times show that although EBD runs slower than FI, it is sufficiently fast to be applicable to real-world, high-dimensional datasets. Overall, our results indicate that EBD is easy to implement and is sufficiently fast to be practical. Thus, we believe EBD is an effective discretization method that can be useful when applied to high-dimensional biomedical data.

We note that EBD and FI differ in both in the score used for evaluating candidate discretizations and in the search method employed. As a result, the differences in performance of the two methods may be due to the score, the search method, or a combination of the two. A version of FI could be developed that uses dynamic programming to minimize its cost function, namely entropy, in a manner directly parallel to the EBD algorithm that we introduce in this paper. Such a comparison, however, is beyond the scope of the current paper. Moreover, since the FI method was developed and is implemented widely using greedy search, we compared EBD to it rather than to a modified version of FI using dynamic programming search. It would be interesting in future research to evaluate the performance of a dynamic programming version of FI.

## Conclusions

High-dimensional biomedical data obtained from transcriptomic and proteomic studies are often pre-processed for analysis that may include the discretization of continuous variables. Although discretization of continuous variables may result in loss of information, discretization offers several advantages. It broadens the range of data mining methods that can be applied, can reduce the time taken for the data mining methods to run, and can improve the predictive performance of some data mining methods. In addition, the thresholds and intervals produced by discretization have the potential to assist the investigator in selecting biologically meaningful intervals. For example, the intervals selected by discretization for a transcriptomic variable provide a starting point for defining normal, over-, and under-expression for the corresponding gene.

The FI discretization method is a popular discretization method that is used in a wide range of domains. While it is computationally efficient, it is not guaranteed to find the optimal discretization for a predictor variable. We have developed a Bayesian discretization method called EBD that is guaranteed to find the optimal discretization (i.e., the discretization with the highest Bayesian score) and is also sufficiently computationally efficient to be applicable to high-dimensional biomedical data.

## Methods

### Biomedical Datasets

The performance of EBD was evaluated on a total of 24 datasets that included 21 publicly available transcriptomic datasets and two publicly available proteomic datasets that were acquired on the Surface Enhanced Laser/Desorption Ionization Time of Flight (SELDI-TOF) mass spectrometry platform. Also included was a University of Pittsburgh proteomic dataset that contains diagnostic data on patients with Amyotrophic Lateral Sclerosis; this data were acquired on the SELDI-TOF platform [17]. The 24 datasets along with their types, number of instances, number of variables, and the majority target value proportions are given in Table 1. The 23 publicly available datasets used in our experiments have been extensively studied in prior investigations [17-34].

### Additional Details about the EBD Algorithm

In this section, we first provide additional details about the *Prior *probability function that is used by EBD. Next, we discuss details of the EBD pseudocode that appears in Figure 1.

Let *D *be a dataset of *n *instances consisting of the list ((*X*_1_, *Z*_1_), (*X*_2_, *Z*_2_), ..., (*X*_*k*_, *Z*_*k*_), ..., (*X*_*n*_, *Z*_*n*_)) that is sorted in ascending order of *X*_*k*_, where *X*_*k *_is a real value of the predictor variable and *Z*_*k *_is the associated integer value of the target variable. Let *λ *be the mean of a Poisson distribution that represents the expected number of cut points between *X*_1 _and *X*_*n *_in discretizing *X *to predict *Z*. Note that zero, one, or more than one cut points can occur between any two consecutive values of *X *in the training set. Let *Prior*(*k*) be the prior probability of there being at least one cut point between values *X*_*k *_and *X*_*k *+ 1 _in the training set. For *k *from 1 to *n*-1, we define the EBD *Prior *function as follows:(10)

where, *d*(*a, b*) = *X*_*b *_- *X*_*a *_represents the distance between the two values *X*_*a *_and *X*_*b *_of *X*, and *X*_*b *_is greater than *X*_*a*_. When *k *= 0 and *k *= *n*, boundary conditions occur. We need an interval below the lowest value of *X *in the training set and above the highest value. Thus, we define *Prior*(0) = 1, which corresponds to the lowest interval, and *Prior*(*n*) = 1, which corresponds to the highest interval.

The EBD pseudocode shown in Figure 1 works as follows. Consider finding the optimal discretization of the subsequence *S*_1, *a *_for *a *being some value between 1 and *n*.^3 ^Assume we have already found the highest scoring discretization of *X *for each of the subsequences *S*_1,1_, *S*_1,2_, ..., *S*_1,*a*-1_. Let *V*_1_, *V*_2_, ..., *V*_*a*-1 _denote the respective scores of these optimal discretizations. Let *Score*_*i *_be the score of subsequence *S*_*i, a *_when it is considered as a single interval, that is, it has no internal cut points; this term is denoted as the variable *Score_ba *in Figure 1. For all *b *from *a *to 1, EBD computes *V*_*b *- 1 _× *Score_ba*, which is the score for the highest scoring discretization of *S*_1, *a *_that includes *S*_*b, a *_as a single interval. Since this score is derived from two other scores, we call it a *composite score*. The fact that this composite score is a product of two scores follows from the decomposition of the scoring measure we are using, as given by Equation 9. In particular, both the prior and the marginal likelihood components of that score are decomposable. Over all *b*, EBD chooses the maximum composite score, which corresponds to the optimal discretization of *S*_1, *a*_; this score is stored in *V*_*a*_. By repeating this process for *a *from 1 to *n*, EBD derives the optimal discretization of *S*_1, *n*_, which is our overall goal.

Several lines of the pseudocode in Figure 1 deserve comments. Line 8 incrementally builds a frequency (count) distribution for the target variable, as the subsequence *S*_*b, a *_is extended. Line 11 determines if a better discretization has been found for the subsequence *S*_1, *a*_. If so, the new (higher) score and its corresponding discretization are stored in *V*_*a *_and *T*_*a*_, respectively. Line 15 incrementally updates *P *to maintain a prior that is consistent with there being no cut points in the subsequence *S*_*b a*_.

We can obtain the time complexity of EBD as follows. The statements in lines 1 and 2 clearly require *O*(1) run time. The outer loop, which starts at line 3, executes *n *times. In that loop lines 3-5 require *O*(1) time per execution, and line 6 requires *O*(*J*) time per execution, where *J *is the number of values of the target variable. Thus, the statements in the outer loop require a total of *O*(*J*·*n*) time. The inner loop, which starts at line 7, loops *O*(*n*^2^) times. In it lines 8 and 9 require *O*(*J*) time, and the remaining lines require *O*(1) time. Thus, the statements in the inner loop require a total of *O*(*J*·*n*^2^) of run time.^4 ^Therefore, the overall time complexity of EBD is *O*(*J*·*n*^2^). Assuming there is an upper bound on the value of *J*, then the complexity of EBD is simply *O*(*n*^2^).

The numbers computed within EBD can become very small. Thus, it is most practical to use logarithmic arithmetic. A logarithmic version of EBD, called lnEBD, is given in Additional file 1.

### Discretization and Classification

For the FI discretization method, we used the implementation in the Waikato Environment for Knowledge Acquisition (WEKA) version 3.5.6 [35]. We implemented the EBD discretization method in Java so that it can be used in conjunction with WEKA. For our experiments, we used the J4.8 classifier (which is WEKA's implementation of C4.5) and the naïve Bayes classifier as implemented in WEKA. Given an instance for which the target value is to be predicted, both classifiers compute the probability distribution over the target values. In our evaluation, the distribution over the target values was used directly; if a single target value was required, the target variable was assigned the value that had the highest probability.

### Evaluation Measures

We conducted experiments for the EBD and FI discretization methods using 10 × 10 cross-validation. The discretization methods were evaluated on the following five measures: accuracy, area under the Receiver Operating Characteristic curve (AUC), robustness, stability, and the average number of intervals per variable.

Accuracy is a widely used performance measure for evaluating a classifier and is defined as the proportion of correct predictions of the target made by the classifier relative to the number of test instances (samples). The AUC is another commonly used discriminative measure for evaluating classifiers. For a binary classifier, the AUC can be interpreted as the probability that the classifier will assign a higher score to a randomly chosen instance that has a positive target value than it will to a randomly chosen instance with a negative target value. For datasets in which the target takes more than two values, we used the method described by Hand and Till [36] for computing the AUC.

Robustness is defined as the ratio of the accuracy on the test dataset to that on the training dataset expressed as a percentage [5]. It assesses the degree of overfitting of a discretization method.

Stability measures the sensitivity of a variable selection method to differences in training datasets, and it quantifies how different training datasets affect the variables being selected. Discretization can be viewed as a variable selection method, in that variables with a non-trivial discretization are selected while variables with a trivial discretization are discarded when the discretized variables are used in learning a classifier. A variable has a trivial discretization if it is discretized to a single interval (i.e., has no cut points) while it has a non-trivial discretization if it is discretized to more than one interval (i.e., has at least one cut-point).

We used a stability measure that is an extension of the measure developed by Kuncheva [37]. To compute stability, first a similarity measure is defined for two sets of variables that, for example, would be obtained from the application of a discretization method to two training datasets on the same variables. Given two sets of selected variables, ***v***_*i *_and ***v***_*j*_, the similarity score we used is given by the following equation:(11)

where, *k*_*i *_is the number of variables in ***v***_*i*_, *k*_*j *_is the number of variables in ***v***_*j*_, *r *is the number of variables that are present in both ***v***_*i *_and ***v***_*j*_, *n *is the total number of variables, min(*k*_*i*_, *k*_*j*_) is the smaller of *k*_*i *_or *k*_*j *_and represents the largest value *r *can attain, and  is the expected value of *r *that is obtained by modeling *r *as a random variable with a hypergeometric distribution. This similarity measure computes the degree of commonality between two sets with an arbitrary number of variables, and it varies between -1 and 1 with 0 indicating that the number of variables common to the two sets can be obtained simply by random selection of *k*_*i *_or *k*_*j *_variables from *n *variables, and 1 indicating that the two sets are contain the same variables. When ***v***_*i *_or ***v***_*j *_or both have no variables, or both ***v***_*i *_and ***v***_*j *_contain all predictor variables, *Sim*(***v***_*i*_, ***v***_*j*_) is undefined, and we assume the value of the similarity measure to be 0.

### Experimental Methods

In performing cross validation, each training set (fold) contains a set of variables that are assigned one or more cutpoints; we can consider these as the selected predictor variables for that fold. We would like to measure how similar are the selected variables among all the training folds. For a single run of 10-fold cross validation, the similarity scores of all possible pairs of folds are calculated using Equation 11. With 10-fold cross validation, there are 45 pairs of folds, and stability is computed as the average similarity over all these pairs. For the ten runs of 10-fold cross-validation, we averaged the stability scores obtained from the ten runs to obtain an overall stability score. The stability score varies between -1 and 1; a better discretization method will be more stable and hence have a higher score.

For comparing the performance of the discretization methods, we used the Wilcoxon paired samples signed rank test. This is a non-parametric procedure concerning a set of paired values from two samples that tests the hypothesis that the population medians of the samples are the same [38]. In evaluating discretization methods, it is used to test whether two such methods differ significantly in performance on a specified evaluation measure.

## Authors' contributions

JLL developed the computer programs, performed the experiments, and drafted the manuscript. GFC and SV designed the EBD method and helped to draft and revise the manuscript. VG assisted JLL in obtaining the biomedical datasets and in the design of the experiments. SV helped JLL in the selection of the evaluation measures. All authors read and approved the final manuscript.

## Endnotes

^1 ^This is based on a search with the phrase "Fayyad and Irani's discretization" that we performed on December 24, 2010.

^2 ^However, in general we can use background knowledge and belief to set the values of the *α*_*ij*_.

^3 ^Technically, we should use the term *n' *here, as it is defined in Figure 1, but we use *n *for simplicity of notation.

^4 ^We note that line 13 requires some care in its implementation to achieve *O*(1) time complexity, but it can be done by using an appropriate data structure. Also, the *MarginalLikelihood *function requires computing factorials from 1! to as high as (*J*-1 + *n*)!; these factorials can be precomputed in *O*(*n*) time and stored for use in the *MarginalLikelihood *function.

## Supplementary Material

Additional file 1 **Logarithmic Version of EBD**. Contains pseudocode for a logarithmic version of EBD. Click here for file
