# Supplementary material for: Application of an efficient Bayesian discretization method to biomedical data
Source: BMC Bioinformatics. 2011 Jul 28;12:309. doi: 10.1186/1471-2105-12-309 (PMC3162539; doi:10.1186/1471-2105-12-309)
Supplement: Additional file 1 — Logarithmic Version of EBD. Contains pseudocode for a logarithmic version of EBD. [file 1471-2105-12-309-S1.DOC]

# A Logarithmic Version of EBD

This section describes a straightforward version of EBD that uses logarithmic arithmetic to deal computationally with real numbers that are extremely small. In the pseudocode below, a prefix “*ln*” attached to a variable or function name is used simply as a naming convention; it does not mean a function call to the *ln* function. When “ln” appears unitalicized in the pseudocode, it does, however, denote the natural logarithm function.

**Algorithm** *lnEBD* /* a logarithmic version of EBD */

**Input**: Dataset *D* and Poisson parameter *λ*.

**Output**: An optimal Bayesian discretization of variable *X* in *D*.

1. *lnV*_0_ := 0;

2. *T*_0_ := {};

3. for *a* := 1 to *n’*

4. *lnP* :=  *lnPrior*(*a*);

5. *lnV_a_* := *-∞;*

6. *U* := (0, 0, ..., 0);

7. for *b* := *a* downto 1

8. *U* := *U* + *W_b_*; /* element-wise addition */

9. *lnML* := *lnMarginalLikelihood*(*U*);

10. *lnScore_ba* := *lnP* + *lnML*;

11. if *lnV_b_*_-1_ + *lnScore_ba* > *lnV_a_*

12. then

13. *T_a_* := *T_b_*_-1_ {*S_b_*, *_a_*};

14. *lnV_a_* := *lnV_b_*_-1_ + *lnScore_ba*;

15. *lnP* := *lnP* + ln(1 – *Prior*(*b*-1));

16. return *T_n’_*

The functions that appear in *lnEBD* are defined below.

*lnPrior*(*a*) is defined as follows:

if *a* = 0 or *a* = *n’* then return 0 else return ln$(1-e^{-\lambda\cdot\frac{d(a,a+1)}{d(1,n')}})$.

*lnMarginalLikelihood*(*U*) is defined as follows:

*lnMarginalLikelihood*(*U*) := $\text{lnfact}(J-1) -\text{lnfact}(J-1+\sum_{j=1}^{J} U_{j}) +\sum_{j=1}^{J} \text{lnfact(}U_{j})$,

where *lnfact*(*w*) is a function that returns ln(*w*!). In the implementation that follows, we assume that these function values are stored in an array that is also called *lnfact*, which we distinguish using square brackets. We can efficiently construct this array using the following iterative method:

*lnfact*[0] := 0;

for *w* := 1 to *n’* + (*J*-1)

*lnfact*[*w*] := *lnfact*[*w*-1] + ln[*w*];
